# Experimental and numerical analysis of the thermal performance of pebble solar thermal collector

**DOI:** 10.1016/j.heliyon.2024.e24218

**Published:** 2024-01-12

**Authors:** N. Channa Keshava Naik, R. Krishna Priya, Ümit Ağbulut, Ali Etem Gürel, Saboor Shaik, Ali Nasser Alzaed, Mamdooh Alwetaishi, Ahmad Aziz Alahmadi

**Affiliations:** a Department of Mechanical Engineering, BGS College of Engineering and Technology, (Affiliated to Visvesvaraya Technological University, Belgaum), Bengaluru, 560086, Karnataka, India; b University of Technology and Applied Sciences, College of Engineering and Technology, Musandam, Engineering Department, Sultanate of Oman; c Department of Mechanical Engineering, Mechanical Engineering Faculty, Yildiz Technical University, Istanbul, 34349, Türkiye; d Department of Electricity and Energy, Düzce Vocational School, Düzce University, 81010, Düzce, Türkiye; e School of Mechanical Engineering, Vellore Institute of Technology Vellore, 632014, Tamil Nadu, India; f Department of Architecture Engineering, College of Engineering, Taif University, Taif, 21944, Saudi Arabia; g Department of Civil Engineering, College of Engineering, Taif University, Taif, 21944, Saudi Arabia; h Department of Electrical Engineering, College of Engineering, Taif University, Taif, 21944, Saudi Arabia

**Keywords:** Pebbles, Flat plate collector, Heat gain enhancement, Thermal efficiency, CFD

## Abstract

In this work, pebbles of higher specific heat than the conventional absorber materials like aluminium or copper are proposed as a absorber in the solar flat plate collector. The proposed collector are integrated into the building design and constructed with masonry. Tests were conducted by varying the operating parameters which influence its performance, like the flow rate of the heat-absorbing medium, and the tilt of the collector using both coated and uncoated pebbles. The maximum temperature difference that could be measured for a conventional absorber was approximately 8 °C for a flow rate of 0.6 L/min. While for a coated and uncoated absorber, it was 7 °C and 5.5 °C respectively. This difference decreased with an increase in flow rates from 0.6 L/min to 1.2 L/min. For all the flow rates, it was observed that the average difference in efficiency between the coated and the conventional absorber collector is 5.82 %, while the difference between the coated and uncoated absorber collector is 15.68 %. Thus, it is very much evident that by replacing the conventional absorber with the proposed coated pebble absorber, the overall loss in efficiency is just 5.82 %, but the advantages are enormous. Along with the experimental study, numerical analysis was also carried out with CFD modeling. The numerical results agreed well with experimental results with the least error. Therefore, CFD simulation can be further used to optimize the design of the collector.

## 1 Introduction

The environment has been heavily contaminated as a result of the emissions of various gases caused by the burning of fossil fuels, which are expensive and widely used to supply the world's energy demands [1]. Renewable energy sources have the potential to replace conventional fuels in four different areas: electricity production, transportation, hot water generation and off-grid power supply [2]. Solar energy has the greatest potential in India when compared to other renewable energy sources. Clear, bright weather is observed roughly 200–250 days per year in most places in India. The yearly radiation fluctuates between 1500 and 2500 kWh/m^2^, which is equivalent to that received in tropical and sub-tropical areas. Solar energy is an easily accessible source of energy for addressing long-term energy issues. When compared to other renewable energy sources, the solar energy sector is undoubtedly the suitable option for meeting future energy requirements [3,4]. For several reasons, solar energy may be the greatest alternative for the future world. The sun produces a total of 3.8 × 10^23^ kW of solar energy, of which the earth absorbs around 1.8 × 10^14^ kW [5]. Solar energy, according to research, may satisfy global energy demand since it is abundant in nature [6]. Secondly, because it is renewable and has a better production efficiency than other energy sources, it is a potential source of energy for the entire world [7]. Solar energy collectors are a type of heat exchanger that transfers solar energy into the internal energy of the transport medium. Any solar system's most crucial component is the solar thermal collector. The commercial building sector is the major area for SWH. Various government programmes are already in place to boost the use of solar energy systems in usual domestic applications. Though, implemented programmes have the goal to accomplish 25–35 % of the prospective uses in future [8]. Solar water heating systems are very popular because water heating accounts for 35–45 % of a family's total electricity bill. Solar energy can help a family save up to 90 % on their water heating costs. The system is capable of meeting all of our summer heating requirements [9]. The most significant component of photothermal conversion technology is the flat plate collector (FPC). This type of stationary collector is commonly used for low to medium-temperature heating applications, such as domestic hot water and low-temperature industrial applications. The FPCs include stationary collectors that require no solar tracking system. Temperature readings at various points of the collector may be used to estimate the dispersion of heat flow. An FPC consists of absorber plates, absorber tubes, glazing covers, inlet and outlet valves, insulation layers and auxiliaries, etc [10,11]. In order to absorb maximum heat, the absorber plate in the collector is normally coated with a blackened surface; nevertheless, several combinations of colour coatings have been presented in the literature [12,13]. The top layer of required selective surfaces is usually thin, and they have a high production cost due to their strong optical performance [14].

Jaisankar et al. [15] found that solar water heating is the most effective method for converting direct incident solar energy into heat energy. When compared to solar electrical direct conversion systems, which have only 18 % efficiency, solar thermal conversion has an efficiency of about 65 %. SangramPatil et al. [16] developed a 2 m × 1 m concrete solar thermal collector, and tested it over several months at varying flow rates. The collected hot water's average temperature during the rainy season was 48 °C–58 °C (49 °C–60 °C in the winter and 57 °C–81 °C in the summer). As a result, they came to the conclusion that the concrete collector is enough for supplying an adequate quantity of hot water for domestic purposes. Hirasawa et al. [17] observed the decrease in convection heat losses from solar thermal collectors when highly-porous Pebbles stones are placed over the collector plate. It was noticed that the total reduction of convection heat loss was only 8% when the average temperature of the collector was 98 °C. Himangshu Bhowmik et al. [18] introduced reflectors (booster mirrors) to enhance the solar collector's overall performance. It was observed that the collector efficiency is 49% without a reflector (booster mirror) and 65% with a reflector. By combining the flat plate solar collector with the reflector, the overall efficiency of the collector is boosted by around 12 %. Xiao-Yu et al. [19] developed Ceramic solar collectors from ceramic and vanadium black ceramic. The solar absorber coating is made of vanadium black ceramic, which has a consistent solar absorbance value of 0.94–0.98. Ceramics have a solar absorption coefficient of 0.94–0.98 and no attenuation. Tabaei et al. [20] found the greatest collector effectiveness at a 14.6° inclination angle from the horizontal surface. The volume flow rate of water was 0.4 L per minute. P.I. Cooper et al. [21] explored the effect of FPC tilt on heat loss. They analyzed the top heat loss coefficient for various plates, wind speeds and ambient temperatures at 0–90° tilt angles and found that the top heat loss co-efficient value decreased from 44 to 90° tilt angles. M.D.T. Afandie et al. [22] developed DVNR 7018 (dynamically vulcanized natural rubber), EPDM (ethylene propylene diene monomer) and DVNR 7018/stainless steel composite as absorber materials for solar collectors, and found that EPDM collectors have slightly better thermal performance than TPNR (thermoplastic natural rubber) collectors. Wilhelm et al. [23] looked at a variety of materials for high temperature and low cost solar collectors. Chlorinated polyvinyl chloride, fluoroplastics, polysulfide, and silicon's were among the materials studied. The authors also proposed installing the collector with a tilt angle of 14° determined for winter solar conditions to prevent stagnation effects. Shukla et al. [24] conducted a comprehensive investigation on the usage of phase change material (PCM) in solar water heating systems (SWHs). Only basic designs of PCM-based SWHs were found to be accessible. C.O. Frank et al. [25] recent breakthroughs in polymer technology have resulted in the creation of acceptable materials i.e. Ethylene propylene diene monomer which is petroleum-based product for solar collectors that can resist long exposure to solar insolation. Kanimozhi B et al. [26] conducted a single-phase solar-powered desalination experiment using a porous media such as pebble stones to collect heat energy and transport it to the working fluids. Solar desalination with porous medium has a 67.9% efficiency compared to 55.2% without porous medium. Hanchen et al. [27] used a packed bed of pebble stones to carry out a heat transfer study of high-temperature thermal storage medium, which was confirmed experimentally for steady heat inflow. The numerical solutions for charging and discharging cycles with a constant heat intake were derived using a transient one-dimensional two-phase energy conservation equation. M.A. Amraoui et al. [28] used computational fluid dynamics (CFD) tool to simulate the solar air collector in order to better understand the heat transfer capabilities. The insertion of proper baffles in solar air collectors enhances the efficiency with rise in temperature. Lingayat et al. [29] used ANSYS FLUENT V16 simulation software to simulate the problem domain and create a 2D numerical model to investigate heat transmission and fluid dynamics processes inside a solar thermal collector of an indirect type solar dryer (ITSD) with square roughness on the absorber plate. In order to improve the performance of a solar still, Hitesh Panchal et al. [30] looked into the effects of using evacuated tubes solar collector, perforated fins, and pebbles. The findings show that the time for the DO peak shifts from 1 to 3 p.m. on a sample day from the six months of experiments, which was in February 2019. Daliran and Ajabshirchi investigated the thermal performance of the SAC both with and without fins [31]. The maximum temperature and thermal efficiency determined by the experimental and theoretical studies, respectively, were 74.5C and 21 % and 83C and 22 %. According to Hosseini et al. [32], the SAH with rectangular fins has thermal efficiencies of 12.5 % and 5.5 % higher than those with elliptical and triangle-shaped fins. Investigations into the performance of SAHs with conical surfaces revealed that the maximum average thermal and energy efficiency of solar collectors with conical surface absorber plates was 74.6 % at 0.1 kg/s mass flow rates and 19.3 % at 0.04 kg/s mass flow rates. Utilising a conical surface area increased airflow obstruction, which increased the turbulent effect and increased the convective heat transfer coefficient [33]. Four flat-plate solar collectors with different channel geometries were simulated by Ambarish Maji et al. [34] using CFD. The investigation focused on how channel turns and geometry affect collector performance. Among the models tested, Model 3, which has more turns while keeping the pipe geometry constant, stands out as the best design. In a study by D.L. Zhao et al. [35] a solar air heating system for a 3319 m^2^ building area in China is modeled using TRNSYS. The system uses a pebble bed and water storage to store heat and has a number of operating modes, including nighttime, auxiliary source, and solar collector and storage bed heating. The system produces an average annual solar fraction of 53.04 % and meets 32.8 % of winter thermal energy demand and 84.6 % of non-heating season energy consumption, according to the validated model.

Nurril et al. [36] analyzed the heat-transfer augmentation technique employing vibration and its potential for FPSC. They also studied new techniques to improve FPSC thermal performance. The author listed ten unique techniques for improving the thermal performance of FPSCs like the use of nanofluid, absorber coatings, heat loss reduction, PCM, thermal performance enhancers, modifying the design of FPSCs, turbulators, mini and micro channels, and polymer materials. Heat-transfer enhancement using vibration technique on ETSC suggests a potential improvement in heat-transfer efficiency up to 78 %. ii) Heat-transfer enhancement using vibration method provides a pressure amplitude that encourages flow alteration and fluid mixing. Seyed Ali Sakhaei et al. [37] studied the impact of using nanofluids as working fluids and passive heat transfer improvement techniques. The found that the most efficient methods for enhancing heat transfer involve the use of turbulators in FPCs and nanofluids as working fluids. Furthermore, when the modification in the external variables during tests, such as the intensity of solar irradiation, is undeniable, the dynamic models are utilized to investigate the dynamic behavior of solar collectors. Harish kumar Sharma et al. [38] examined theoretically and experimentally the impact of translucent insulating materials between top glazing and absorber surface for integrated solar water heating and storage systems. Observations show that, in comparison to parallel and non-TIM systems, TIM perpendicular layout offers improved thermal and storage efficiency. Djemaa Guerraiche et al. [39] investigated the thermal performance of integrating a PCM in a CPC concentrating system. As PCM, they employed RT42-graphite and myristic acid. It was placed within the outer tube, and water was allowed to flow inside the inner concentric tube. The system showed an increase in thermal efficiency for a longer period of time for the two PCMs under consideration, with the output water temperature differential for both PCMs increasing by roughly 12 °C. Sujit Kumar Verma et al. [40] proposed a flat plate solar collector with a single spiral-shaped collector tube rather than many riser tubes coupled with headers. It has been shown that the efficiency of the solar collector have improved significantly while maintaining all other parameters at their standard levels. The improvement in thermal efficiency obtained during forced mode of testing was 21.94 % greater than that of the traditional flat plate collector design. Ahmad Zarei et al. [41] used a unique solar-powered combined cycle. The suggested system offers power, heating, cooling (for refrigeration and freezing needs), and heating for household applications. It also has a booster compressor to improve system performance. M. Sheikholeslami et al. [42] provided a thorough overview of recent advancements, techniques, important economic factors, the importance of solar water heating and the difficulties associated with implementing such solar water heating systems. H.M. Teamah et al. [43] examined the integration of PCM and PCM nanocomposite in the FPSC. An In-depth system modeling analysis was also provided. The most significant improvements in PCM thermal conductivity have been seen in metal foams and carbon-based nanocomposites. The heat transfer fluid tubes are said to be the ideal location for PCM incorporation. The addition of PCM ensured that the collector would continue to deliver hot water for a longer length of time. R. Santbergen et al. [44] found that the mono-crystalline PV cell can absorb more light than other varieties of PV cells. A.E. Kabeel et al. [45] developed a solar still with a turbulence system powered by solar PV that increases the production of pure water. This approach is more affordable and generates a higher daily return than traditional stills. The weir-type inclined solar still was the subject of research by Sadineni et al. [46] who reported that it has a daily productivity of 5.5 l/m^2^ and performs better in shallow water. In a similar work, Kumar A. et al. claimed that deterioration and failure mechanisms are crucial for the precise prediction of solar PV system performance. They fully presented different solar PV technology and used mathematical modeling to characterize the solar PV systems [47,48]. A new form of portable thermoelectric solar still (PTSS) was developed by N Rahbar et al. [49], in which a thermoelectric module was utilized to increase the temperature difference between the evaporating and condensing zones. For a period of 12 months, Diamantino et al. [50] looked into the impact of atmospheric conditions on the rate of corrosion of standard samples such carbon steel, zinc, copper, and aluminum. Two outdoor exposure test (OET) sites with urban and industrial atmospheres at a 45 °C southerly angle were used to study absorber specimens. Extremely corrosive metals in terms of south orientation like zinc and copper were added.

An extensive literature survey revealed that no detailed study is done using pebble as an absorber material in a solar flat plate collector. However, there is enough work carried out using pebble as a heat storage medium [[51], [52], [53], [54], [55]]. In our work, the pebbles serve as a heat sink that absorbs the incident solar radiation on the surface of the collector. The pebbles have high thermal conductivity and can efficiently store thermal energy. Using pebbles as a thermal absorber in flat plate collectors provides several advantages over conventional designs. First, it increases the available surface area for heat transfer, which improves the collector's efficiency. The pebbles act as a thermal insulator, thereby reducing heat loss through conduction and radiation. Thirdly, it increases the collector's durability and dependability because the pebbles are resistant to corrosion and can withstand high temperatures. In summary, this approach will enhance the thermal performance of the flat plate collector by increasing the heat transfer rate and reducing heat losses.

There are three most critical features of this project which make it unique and can benefit the tour country to India. One is, by this technology the cost of the solar collector reduces considerably which increases its popularity among the public and this will enhance the customers to opt for this collector. As the thermal properties of the pebbles are better than that of the conventionally used Al and Cu absorbers. The performance of the new collector will be more than that of the conventional collectors. The second feature is the prevention of pollution that would be caused while mining and transporting the ore and conversion of the ore to useable metals used in the conventional collector. Since the metals are completely removed from the collector, the problem of corrosion is also completely eliminated and the subsequent pollution that would be created while cleaning the metals is also prevented resulting in a low carbon footprint. This will also result in zero maintenance cost of the collector thus making it even more popular.

A numerical simulation was also developed to evaluate its performance for varying operating conditions and also its structural stability, to ascertain its design aspects. PASTC is computationally modeled in CATIA V5 modeling software. The meshing of the proposed model was carried out using Hexa and Tetra dominant meshing methods. The meshed model was exported to ANSYS structural V19.2 which is a commercial simulation software for the analysis. The analysis revealed a safe structural design, with an acceptable convergence between the simulated and experimental findings of the working fluid at the collector outlet. There are five sections in the paper. An overview of the introduction is given in Section 1. The materials and methodology used are covered in Section 2. The experimental setup is presented in detail in Section 3 while the numerical aspects is covered in Section 4. The results are thoroughly discussed in Section 5, which is followed by the conclusion.

## 2 Materials

In conventional solar collector, metals like copper or aluminum are extensively used for heat absorption and the chemical treatment used to prevent the corrosion of these metals will adversely lead to pollution. Mining and processing of metal ores causes severe environmental and health-related problems around the world. Mines produce large amounts of waste since the ore is only a small fraction of the total volume of the mined material. In the metal industry, production of Cu, Pb, and Al causes the greatest degradation of environment. Copper mining produces extensive mine wastes and tailings and emissions from primary copper smelters are principally particulate matter and sulfur oxides (SOx). Corrosion maintenance of Cu and Al consumes a lot of energy and pollutes the environment. Steel production produces the maximum greenhouse emissions worldwide, accounting for nearly 7 % of total CO_2_ generated by fossil fuels [56]. The annual CO_2_ manufacture of Al is higher than the of steel and copper (about 40 MT per year) and aluminium production is responsible for around 3 % of global CO_2_ [57]. The quantity of greenhouse gases produced follows closely the trends in embodied energy (Table 1a) though for those metals which require a high component of electrical energy, such as aluminium and copper, the source of electrical energy (coal, hydro, nuclear, etc) has a major impact on the quantities of greenhouse gases produced.

**Table 1:** Global CO_2_ production for primary production of metals (Cu &Al).

Metal	% of total global metal production	Global annual production (Mt)	Tonnes Co_2_ per tonne metal	Global annual energy consumption (GJ)	Global annual CO_2_ (tonnes)	% Global greenhouse gas production
**Copper**	80	15.6	16.16	6.13 × 10^8^	6.0 × 10^7^	1.21
**Aluminium**	100	38	21.81	8 × 10^9^	8.3 × 10^8^	2.9

**Fig. 1:**
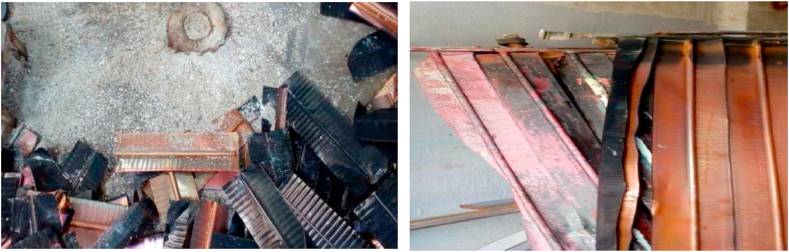
Corroded copper finned tubes.

The samples of corroded copper finned tubes are given Fig. 1. From Table 1 and it is observed that copper and aluminium results in considerable energy consumption and generates pollution.

**Fig. 2:**
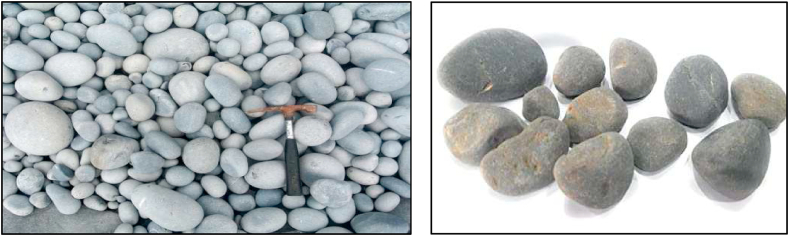
Sedimentary Pebbles [33].

In order to overcome the above-mentioned ill effects of the metals it is proposed to have a metal-free solar collector wherein while constructing the building itself the suggested Pebble Absorber Collector can be built-in on top of the building integrated with its design. This will completely eliminate the use of metals and necessitates maintenance-free operation of the collector that lasts till the life of the building. Thus the proposed project deals with a low carbon, cheap and efficient solar collector making the technology more attractive to common people. A pebble is a clast of rock with a particle size of 5–65 mm. Pebbles are larger than granules (with a diameter of 4–7 mm) and lesser than cobbles (62–250 mm diameter) as represented in Fig. 2. The nature of the source from which the particles were produced is typically indicated by their composition [58]. Pebbles occur in a variety of colors and textures (refer to Fig. 2). Pebbles are typically smooth; however, their smoothness varies depending on how often they come into touch with salt water [59].

Beach Pebbles, Sand-Blasted Pebbles, Stream Pebbles, and Glacial Pebbles are the different types of pebbles discovered [60]. Pebbles are found on the shores of oceans and seas, as well as inland, where ancient waters formerly covered the land [61]. Pebbles used in the present work are collected from Mangalore Sea shore (Mangalore Beach) and free of cost, by following the Eco-friendly methods.

In geology and sedimentology, the Krumbein phi (φ) scale is often used to assess and characterize the grain size distribution of sedimentary particles such as sand, silt, and clay. It enables a systematic and quantitative method of describing the size of sediment particles. It is a logarithmic scale determined using Eq. (1). established by W. C. Krumbein [62,63] in 1934.

(1) $$ɸ=‐\log_{2}\;\frac{D}{D0}$$

where, ɸ is the Krumbein phi scale, D is the diameter of the particle in millimeters, D_0_ is a reference diameter, equal to 1 mm.

### 2.1 Selective solar absorber coatings

Thermal collector's absorber surfaces absorb the majority of incoming solar incident radiation while retaining a significant amount of heat energy through re-radiation from the heated surface [64]. Sol–gel processes may be used to create a wide range of solar absorber thin film. The advantages of such surfaces include their ease of manufacture and possible cost-effectiveness [65]. Acrylic black paint coatings are now a viable option for absorbing surfaces, with benefits such as simplicity of application, cheap cost, commercial availability and durability [66].

**Fig. 3:**
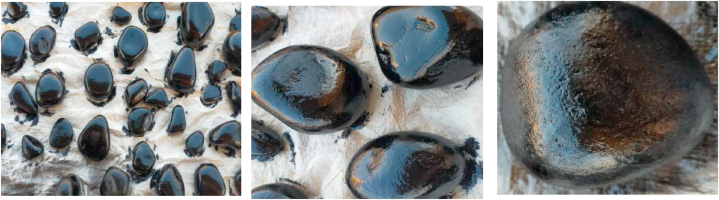
Different Pebble shapes with acrylic black paint coating.

**Fig. 4:**
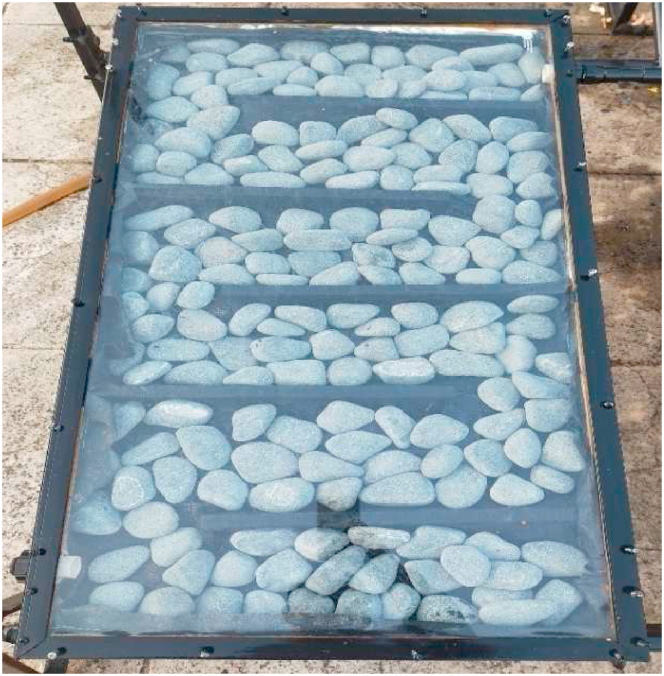
Uncoated pebble collector.

**Fig. 5:**
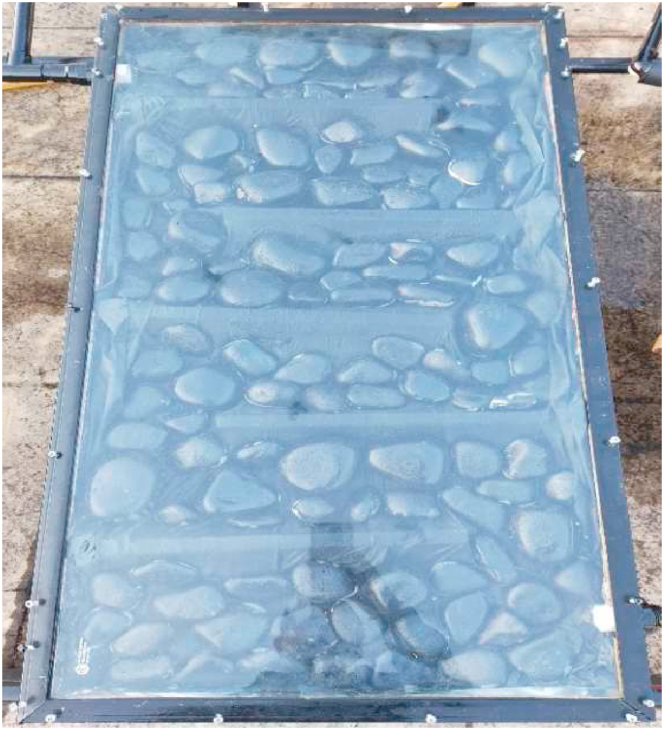
Coated Pebble collector.

As mentioned earlier the study was carried out with both coated and uncoated pebbles in order to ascertain the effectiveness of the coated pebbles (Fig. 2, Fig. 3). The fluid flows from the collector's inlet to the outlet at a consistent velocity and in a zig-zag pattern using wooden baffles, as represented in Fig. 2, Fig. 3. To generate a greenhouse effect and maximize incoming incident radiation by minimizing losses, the collectors were covered with a transparent glass cover with a thickness of 5 mm. The heat from the heated pebble stones is absorbed by the water. Sputtering was originally intended to coat the pebbles, but it was substituted by two methods: dipping and spraying due to technical difficulties. For future use, the stones were dipped in black acrylic paint and dried. The coating was not homogeneous after drying. As a result, the second approach was used to provide a consistent coating. The black acrylic paint was sprayed on and allowed to dry in the second technique and these coated pebbles were utilized as the heat-absorbing medium. As illustrated in Fig. 4, Fig. 5, the collectors in both configurations were of the same size and all were connected to the same single water tank. The studies were carried out to determine heat gain as the flow rate changed.

### 2.2 Methodology

**Fig. 6:**
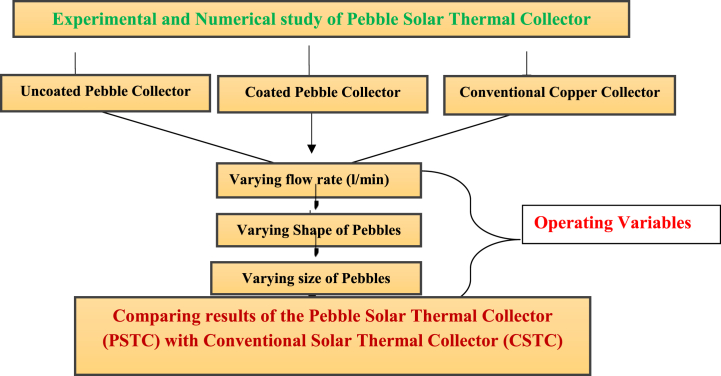
Experimental methodology.

Variation of flow rate through the heat absorbing medium plays a very crucial role in the performance of the collector and hence trials were conducted for different days each by varying the flow rates (0.6 L/min, 0.8 L/min, 0.9 L/min, 1 L/min and 1.2 L/min for six days), where in average results of observed and simulated temperature differences were tabulated. Similarly, the corresponding average heat gain and the average efficiencies are also calculated. The performance of the modified collector (having coated/uncoated pebbles) and the conventional collector are compared for varying size and shape of the pebbles. A numerical simulation was also developed to evaluate its performance for varying operating conditions. As observed from these results, there is no much difference in the observed and numerical values suggesting good agreement between the two. The experimental methodology is shown in Fig. 6 below.

## 3 Experimental set-up

**Fig. 7:**
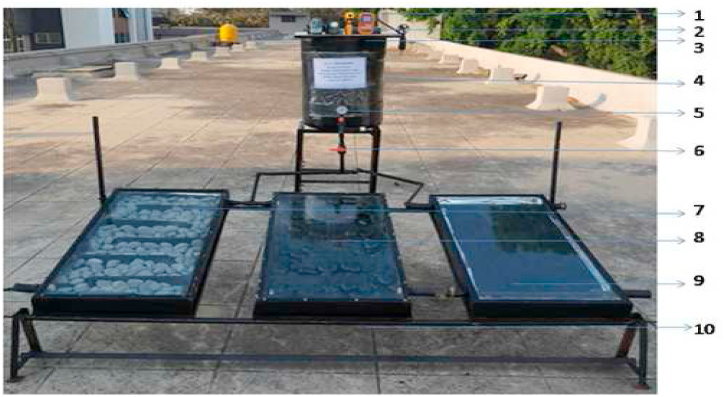
Experimental setup.

In order to investigate the thermal performance and to compare the proposed collector with the conventional collector, under the same environment following three different collector units are fabricated. Experimental rig is given Fig. 7.

Type I: Flat plate collector (FPC) with un-coated pebble absorber collector.

Type II: Flat plate collector (FPC) with coated pebble absorber collector.

Type III: Conventional Flat Plate collector (CFPC) with copper absorber collector.

1. Digital display 2. Digital Anemometer 3. Storage Water tank upper lid 4. Storage Tank
5. Flowmeter 6. Flow Control valve 7. ncoated pebble collector 8. Coated pebble collector 9. Conventional collector 10. Fixed frame.

In order to ensure a common environment for all the three collectors, they were mounted on the same framework and were connected to a common storage tank as shown in Fig. 7. The overall area of PSTC model is 2.16 m^2^ with 0.005 m thick transparent toughened glass plate covering the top of the collector. Rockwool insulator of 0.05 m thickness is placed beneath the absorber. Inlet and outlet ducts are of circular cross-section with a diameter of 0.0508 m. The experimental setup is fixed at one end as shown in Fig. 3a and to vary the tilt angle of collector, the header side is free to move and is provided with a nut and bolt arrangement, with the bottom side permanently fixed to the frame. In the Pebble collector, metal absorbers are replaced by pebble stones and they are arranged on the base of the collector frame and working fluid was allowed to flow over the surface of pebble stones in a zigzag pattern made by positioning wooden baffles in its path in the collector. The zig-zag pattern slows the flow velocity, giving water enough time to absorb solar energy from the pebbles that serve as absorbers. The experimental setup was oriented south facing with an inclination equivalent to the location's latitude (14°) in order to increase the incidence solar radiation. Though the aim of this work is to completely eliminate the use of metals, in the experimental setup, GI sheet had to be used since the setup was temporary, while in the field application this can be avoided by integrating the collector along with the building design and constructing the collector base in masonry construction. Galvanized iron (GI) sheet frame are basically steel sheets that have been coated with zinc. The dimensions of GI sheet are 0.6 m × 1.2 m (for three collectors) with a thickness of 1.2 mm and are used as base frame material in the collector. The pebble collector was constructed such that fluid flows at a consistent velocity and in a zig-zag pattern from the collector's inlet to the outlet. As an alternative to a conventional metal absorber, pebble stone absorbers were placed on the bottom surface, and working fluid was allowed to flow over the pebbles in a zig-zag pattern created by wooden baffles. The thickness of wooden baffles is 0.02 m are used. The term "rockwool" refers to a type of fibre made by spinning or pulling molten minerals (or synthetic minerals such as slag and ceramics). Rockwool provides thermal insulation in the proposed flat plate collector. The overall area of rockwool used in the collector is 1.2 m^2^ with 0.05 m thickness. Tough, Durable and flexible, Strong and Long-Lasting Product. This is also the sheet that separates the pebbles and the insulator. The working fluid is made to flow on this thin polyethylene sheet. The sheet prevents the water from leaking into the insulator section. It also helps to hold the insulating material rigidly. A storage tank with a capacity of 50 L was built 6 feet above the collectors to provide water to collector inlet. Experiments were carried out in Tumkur, Karnataka's Siddaganga Institute of Technology with coordinates: 14° 21′ 18.682″ North, 77° 5′ 5.3760″ East, Latitude 13.4318 and Longitude 77.85.

The Accuracy of the measured Instruments is shown in Table 2.

**Table 2:** Accuracy of the measured Instruments.

	Instruments	Purpose	Specification	Manufacturer Name (Country Name)
01	Digital Anemometer GM 8901 for Air Velocity	To measure the wind speed and the wind direction.	Model: GM8901 Accuracy ±3 %	Hunan Firstrate Sensor Co.,Ltd. India.
02	Digital Luxmeter [LX-101]	To measure radiation intensity on the collector surface.	Model: LX-101 Accuracy (23 ± 5 °C)	LISUN Instruments Limited, India.
03	Digital fluid flow meter	To measure the flow rate of water	Model: Konark MultiJet Accuracy ±2 % 30 ltr/hr Capacity	ATO Flow Meters, China.
04	Thermocouples	To measure the Inlet and outlet working fluid temperature	Model: T-type Thermocouple Accuracy ±0.5 °C	Tempsens Instruments (I) Pvt. Ltd. India

Standard uncertainties in experimental data are determined by taking into account Type A and Type B uncertainties. According to the recommendation of ISO VIM (1995), the former are the uncertainties determined by statistical means while the latter are determined by other means.

**Table:** 

Type An Uncertainties in measurements		Type B Uncertainties in measurements	
Quantity	Accuracy	Quantity	Type B uncertainty
Water temperature	±0.2 °C	Water temperature	U_B_, T_in_, T_out_ = 0.06 °C
Flowrate	±1.0 °C	Flowrate	U_B,m_ = 0.0058 m
Ambient air temperature Ta	±0.5 °C	Ambient air temperature Ta	T_a_ = 0.29 °C
Temperature difference ΔT	±0.1 °C	Temperature difference ΔT	ΔT = 0.06 °C
Collector aperture	±0.1 °C	Collector aperture	Ac = 4.5 *10^−4^ Ac
Solar irradiance	Pyranometer Class 2	Solar irradiance	U_B,G_ = 4 W/m^2^

The uncertainty which is determined statistically (Type An uncertainty), which represents the deviation of the measured value during sampling of data will be used for the determination of one point, the uncertainty of the measuring instrument (Type B uncertainty), is a characteristic feature of the instrument itself. All measurements satisfy the requirements of the standard, using calibrated measuring instruments.

## 4 Numerical simulation of pebble solar thermal collector [PSTC]

A numerical simulation of PSTC is carried out to evaluate its thermal performance for varying operating conditions and also to ascertain its structural stability. The modelling is carried out using CATIA V5 modelling software. The data for the modelling, such as ambient temperature (T_a_), inlet water temperature (T_i_) and incident solar insolation (I) was derived from a few preliminary experimental trials. Next the meshing of the proposed model was done using Hexa and Tetra dominant meshing methods. The meshed model was exported to ANSYS structural V19.2 software for analysis. A convection boundary condition is applied at the bottom of the absorber plate, and a constant heat flux (solar radiation) is applied at the top of the plate. The h_b_ (convective heat transfer coefficient at the bottom) is expressed by Eq. (2) [67]. This paper extends the multipoint flux-approximation (MPFA) control-volume method to quadrilateral grids for which the adjacent cells do not necessarily share corners. Examples are grids with faults and locally refined grids.

(2) $$h_{b}=2:8+3v_{w}$$

The equations governing the flow are given in Eqs (3), (7) [60]:

Continuity equation is given in Eq. (3).

(3) $$\frac{\partial u}{\partial x}+\frac{\partial v}{\partial y}+\frac{\partial w}{\partial z}=0$$

Momentum equations are given in Eqs. (4), (5), (6).

(4) $$\rho u\frac{\partial u}{\partial x}+\rho v\frac{\partial u}{\partial y}+\rho w\frac{\partial u}{\partial z}=-\frac{\partial p}{\partial x}+\mu \left[\frac{\partial^{2}u}{\partial x^{2}}+\frac{\partial^{2}u}{\partial y^{2}}+\frac{\partial^{2}u}{\partial z^{2}}\right]\left(x-\;\text{direction}\;\right)$$

(5) $$\rho u\frac{\partial v}{\partial x}+\rho v\frac{\partial v}{\partial y}+\rho w\frac{\partial v}{\partial z}=-\frac{\partial p}{\partial y}+\mu \left[\frac{\partial^{2}v}{\partial x^{2}}+\frac{\partial^{2}v}{\partial y^{2}}+\frac{\partial^{2}v}{\partial z^{2}}\right]\left(y-\;\text{direction}\;\right)$$

(6) $$\rho u\frac{\partial w}{\partial x}+\rho v\frac{\partial w}{\partial y}+\rho w\frac{\partial w}{\partial z}=-\frac{\partial p}{\partial z}+\mu \left[\frac{\partial^{2}w}{\partial x^{2}}+\frac{\partial^{2}v}{\partial y^{2}}+\frac{\partial^{2}w}{\partial z^{2}}\right]\left(z-\;\text{direction}\;\right)$$

Energy equation is given in Eq. (7).

(7) $$u\frac{\partial T}{\partial x}+v\frac{\partial T}{\partial y}+w\frac{\partial T}{\partial z}=\frac{K}{\rho C_{P}}\left(\frac{\partial^{2}T}{\partial x^{2}}+\frac{\partial^{2}T}{\partial y^{2}}+\frac{\partial^{2}T}{\partial z^{2}}\right)$$

### 4.1 Modelling of the proposed collector

**Fig. 8:**
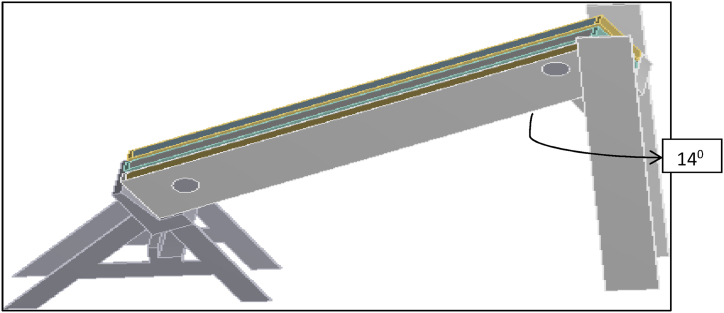
Side view of the 3D isometric model.

**Fig. 9:**
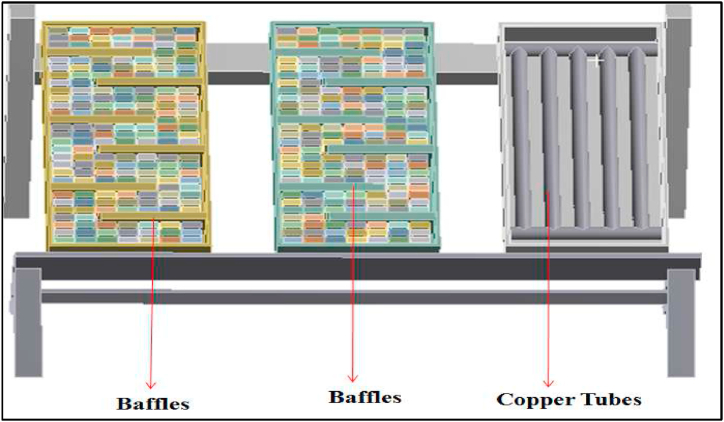
Front view of 3D isometric model.

Modelling of the setup was done using CATIA V5 modelling software. The side view and the front view of 3D isometric model are represented in Fig. 8. and Fig. 9 respectively.

### 4.2 Grid generation

**Fig. 4.11:**
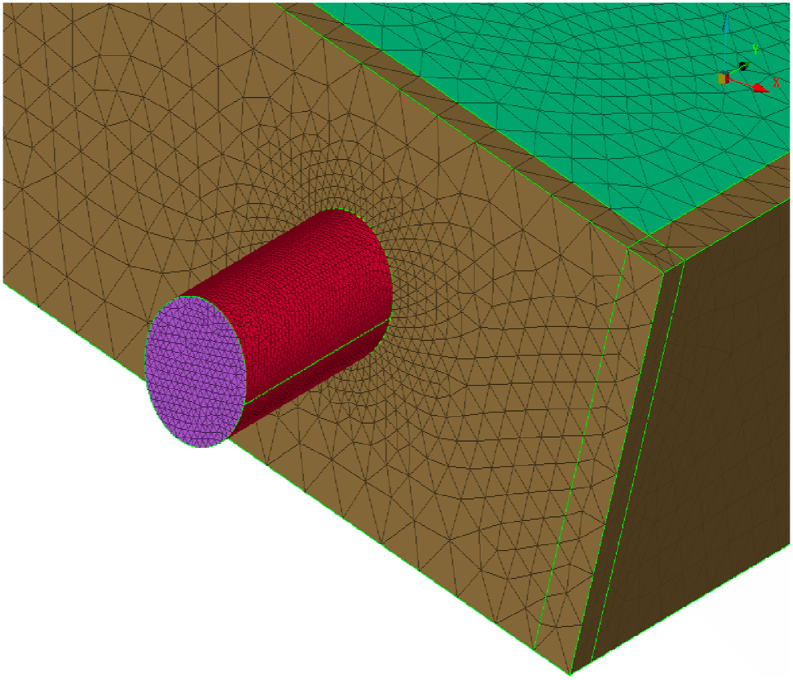
Meshing near the tube.

**Fig. 10:**
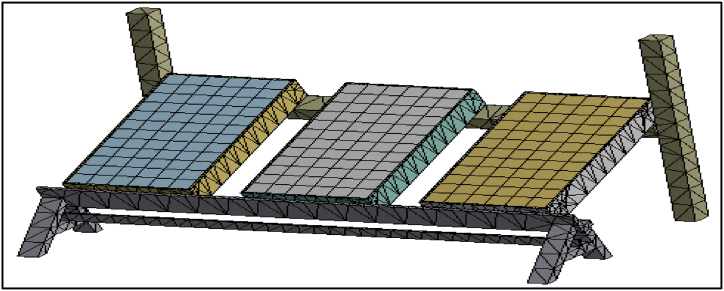
Meshing of the outer structure.

Meshing of the domain was done using ANSYS software, the mesh influences the accuracy, convergence, and speed of the simulation. Here the combinations of hexa and tetra dominant meshing elements (Fig. 10, Fig. 4.11) were used because from these elements the unmeshed regions will mesh automatically and also accuracy level will be high. Meshing resulted in 4,32,891 elements and 11,93,432 nodes. In total 0.17 million surface mesh and 0.68 million of Tetrahedral mesh were generated. In general, a higher-quality mesh yields better results while taking more time to compute.

The next step after creating a mesh is to get ready for grid independence. The number of nodes must be increased in order to create a mesh whose outcomes differ only in scale and are also solvable in a reasonable amount of time. When the mesh is too small, iterative calculations take a very long time and may produce divergent results. The number of components that affect the computing time and the quality of the outcomes are always trade-offs. The mesh is ideal when the results are independent of cell size. We tested the independence of three mesh designs. Table 3 displays the number of nodes used in each design. The same boundary conditions and settings were utilized in each numerical investigation of the three grid's design for each of the three meshes: 1, 2, and 3. By contrasting the temperature difference for the three different meshes, we evaluated how sensitive the numerical findings were to the mesh. Table 3 demonstrates that numerical analysis using meshes 2 and 3 produces the same temperature difference. From this, we can note that the findings become mesh-independent from node number 11,90,900.

**Table 3:** Grid independence test.

Mesh	Nodes	ΔT °C of conventional collector for 0.6 L/min on April 03, 2021.
01	11,90,700	7.1 °C
02	11,90,800	7.8 °C
03	11,90,900	8.0 °C

**Fig. 12:**
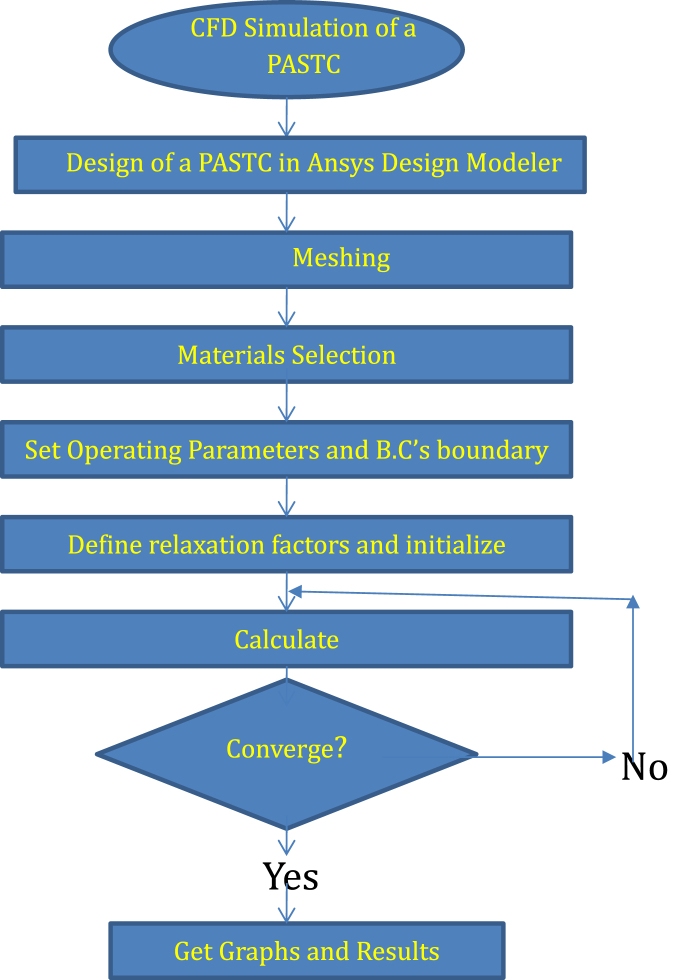
Flow chart for concluding CFD simulation process.

After more grid refinement, the inaccuracy was reduced to less than 0.6 %. There are very few investigations about cross sections of the solar collector, through which the fluid flows, the selected sections were chosen according to thermal analysis. CFD model proposed in the present investigation was validated using the simulation model of Gunjo et al. [68]. The flow chart for conducting the CFD simulation process is shown in Fig. 12.

### 4.3 Theoretical calculation

The thermal performance of the proposed pebble collector (coated and uncoated pebble stones) and conventional copper absorber collector are calculated using the equations proposed by G. D Rai et al. [69] and Shivakumar et al. [70]. Table 4 lists some of the constant values used in the calculations. The overall heat gain by the collector can be calculated using Eq (8).

(8) $$Q_{w}=m_{f}c_{p}\Delta T=m_{f}c_{p}\left(T_{outlet}-T_{inlet}\right)$$

**Table 4:** Constants used in calculation [70].

Parameter	Constant Value
Density of Pebbles (ρ_r_)	1320 kg/m^3^
Density of working fluid (ρ_w_)	1000 kg/m^3^
Density of the rockwool (ρ_r_)	140 kg/m^3^
Specific heat of pebble stones (Cp_p_)	970 J/(kg.K)
Specific heat of water (Cp_w_)	4184 J/(kg.K)
Specific heat of rockwool (Cp_r_)	840 J/(kg.K)
The emissivity of the toughened glass (ε_c_)	0.92

Several factors influence the total efficiency of the collector (η), including the material used, the characteristics of the glass, the design of the absorber and weather conditions.

The collector efficiency (η) is a measure of a flat plate collector's performance. It is defined as the ratio of useable energy gain (Qu) to incident solar energy during a certain time period.

The thermal efficiency of the collector is given in Eq. (9).

(9) $$\eta =Q_{w}/\left(A_{c}I\right)$$

where A_c_ is collector area (m^2^) and I is incident solar radiation (W/m^2^).

### 4.4 Economic analysis

Pebble solar thermal collector is designed for the domestic purpose. So hot water requirement for the domestic purpose is calculated. A 100 L per day capacity system suitable for 4–5 people. Electricity is expensive and is not available due to power cuts in many areas when required for heating water.

One home is considered of five persons. Typical requirement of water for per person per bath per day = 20 L.

Requirement of water for five persons per bath per day = 100 L.

To full fill the requirement of hot water for five persons electric geyser is in use.

Recommended capacity of Geyser to satisfy five persons for bath = 50 L.

A) Specifications of geyser.

**Table:** 

Capacity	Phase	Rated power input (watts)	Hot water output (°C)	Price (Rs)
35 L Single phase	Single phase	3 kW	60 °C	12,000/-

Energy required by Geyser = 3 kWh

Energy required to for heating of water to 60 °C.

Time takes for 3 kW, 35 L Geyser to heat the water to 60 °C = 50 min.

Therefore, energy required to heat the water to 60 °C = 0.666 × 3000 kWh = 1.998 kWh = 1.9 unit Therefore, energy required to heat the water in one day = 1.9 unit per day.

Calculation of energy saving if pebble solar thermal collector is used since average mass flow rate of solar water heater = 0.001 kg/s. So, collection of hot water per day = 33 L,

Requirement of hot water at = 60 °C.

Temperature of water required for the healthy bath = 40 °C.

Average outlet temperature of hot water (T_fo_) for Tumkur = 72.57 °C.

Assume that cold water temperature for all climate zones = 15 °C.

Cold water temperature (T_co_) for Tumkur = 10 °C.

We need to calculate the size of solar water system for all six climate zones of India.

Requirement of water for per person per bath per day = 20 L.

Requirement of water for five persons per bath per day = 100 L.

Therefore for Tumkur to provide 100 L of water, 66.10 L of cold water at 10 °C need to mix with 33.89 L of hot water at 69.73 °C to get 100 L of water at 30 °C. Similarly For Tumkur to provide 100 L of water, 69.21 L of cold water at 10 °C need to mix with 30.61 L of hot water at 74.94 °C to get 100 L of water at 30 °C.

## 5 Results and discussion

### 5.1 Varying flow rate (L/min)

**Fig. 13:**
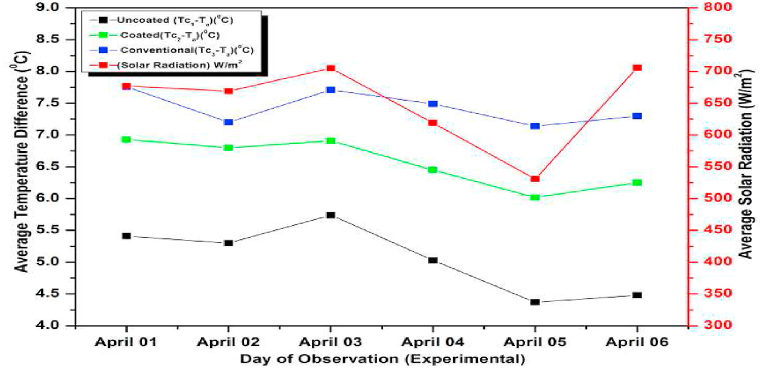
Variation of Day wise Average Temperature Difference (ΔT) (experiment) for 0.6 L/min.

**Fig. 14:**
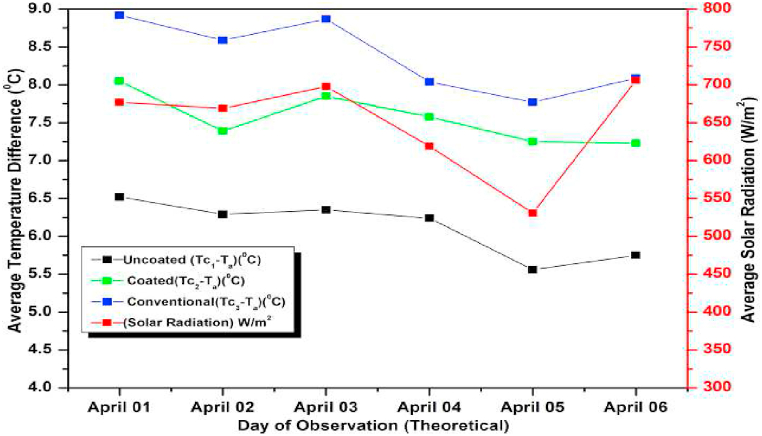
Variation of Day wise Average Temperature Difference (ΔT) (experiment) for 0.6 L/min.

The experiment was conducted for six days from April 01, 2021 to April 06, 2021. The experimental and simulated results for a flow rate of 0.6 L/min for these days along with the measured solar radiations are depicted in Fig. 13, Fig. 14, respectively. Both the graphs are drawn with respect to the temperature difference (ΔT), since the heat gain and collector efficiency mainly depends on the temperature difference. The findings demonstrated that the collector temperature differences increased parallel to the rise in the solar radiation for the same mass flow rate [71]. On April 06, 2021, the authors recorded the highest daily solar radiation of 700 W/m^2^.

The maximum temperature difference that could be measured for a conventional absorber was approximately 8 °C for a flow rate of 0.6 L/min on April 03, 2021. While for a coated and uncoated absorber, it was 7 °C and 5.5 °C respectively. It can also be observed that the temperature differences of the coated collectors are slightly lower than the conventional type for other days also indicating a slight lower efficiency (approximately 6 % less) and heat gain of the coated collectors (calculated using equation (9)) [72]. So low cost coated absorbers can be used instead of high-cost conventional collectors at the slight sacrifice in efficiency. Both in Fig. 13, Fig. 14 the simulated and experimental temperature differences show a small variation suggesting a good agreement between the two. This agreement confirms the accuracy of the CFD analysis presented for the solar collector in the previous section [73].

**Fig. 15:**
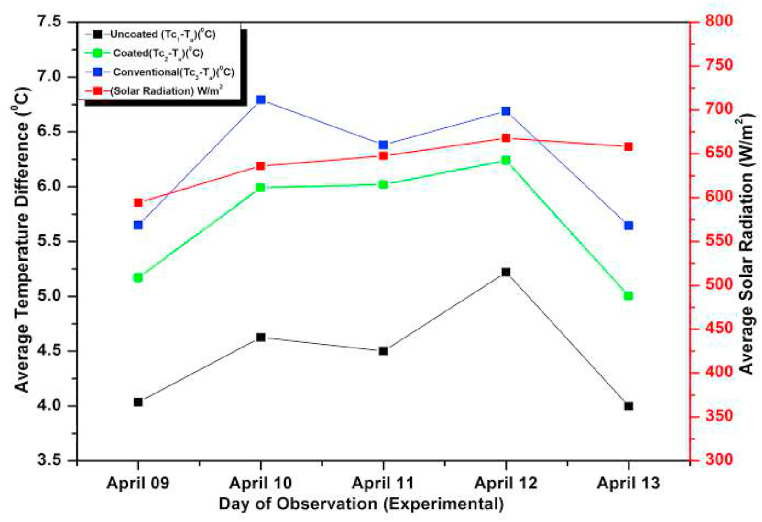
Variation of Day wise Average Temperature (Difference (ΔT) (Observed) for 0.8 L/min).

**Fig. 16:**
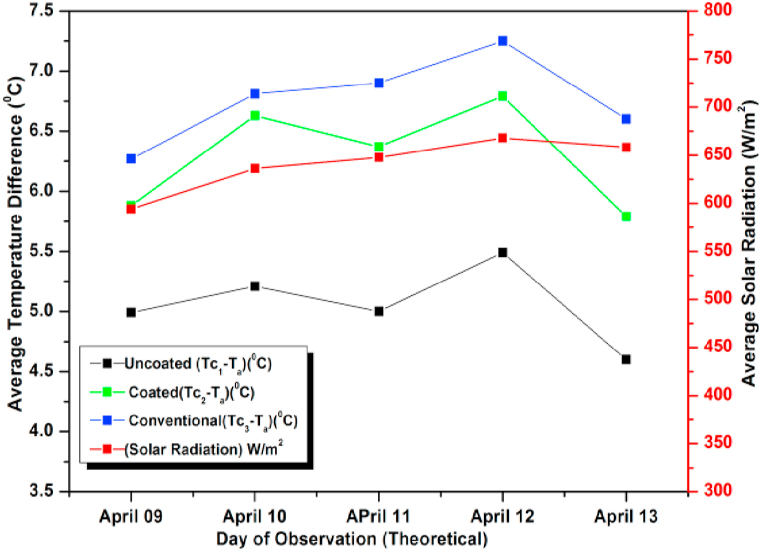
Variation of Day wise Average Temperature (Difference (ΔT) (simulated) for 0.8 L/min).

Fig. 15, Fig. 16 show experimental and numerical plots of average temperature difference (ΔT) w.r.t different days of observations. In these experiments, we increased the mass flow rate from 0.6 L/min to 0.8 L/min. Again, in both the graphs it is evident that the ΔT for the un-coated absorber is minimum while it is maximum for the conventional absorber and the coated absorber lies between the two, suggesting a marginal decrease in performance of the coated pebble absorber compared to a conventional absorber. The higher ΔT and efficiency of the copper absorber solar collector can be attributed to its higher thermal conductivity, which allows it to absorb and transfer heat more efficiently. On the other hand, the coated pebble absorber solar collector has a lower thermal conductivity compared to copper, which may result in a slight decrease in performance. However, it is still a cost-effective and readily available alternative to the conventional copper absorber. For all the days, the ΔT relative error between tests and CFD results was less than 2 %. The variation was because of the assumptions made during numerical analysis and also due to some of the losses that occur during observations which could not be taken into consideration due to their complexity.

**Fig. 17:**
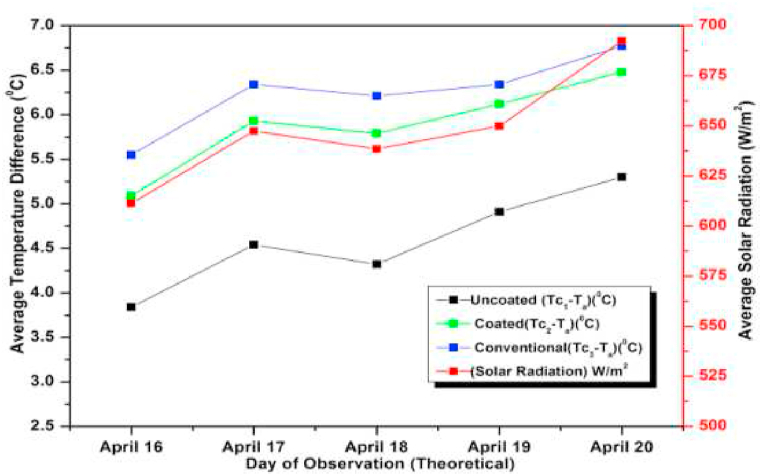
Variation of Day wise Average Temperature Difference (ΔT) (Observed) for 0.9 L/min.

**Fig. 18:**
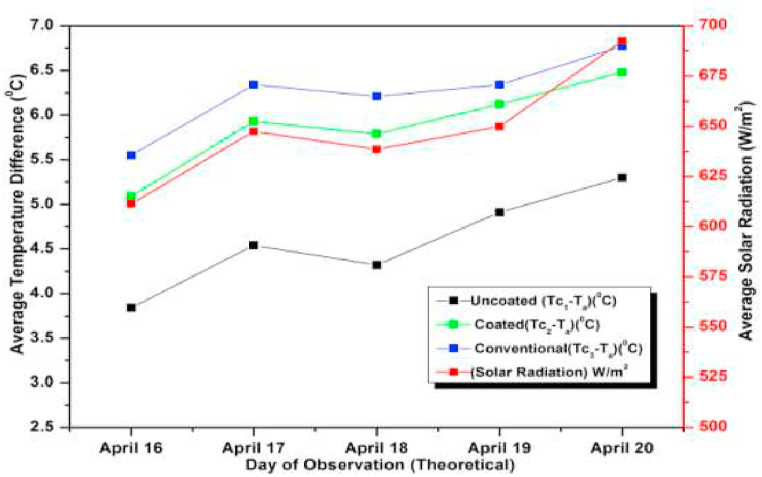
Variation of day-wise average temperature difference (ΔT) (simulated) for 0.9 L/min.

The fluid flow rate was further increased to 0.9 L/min. The experimental and simulated results are shown graphically (Fig. 17, Fig. 18). On April 20, 2021 the incident radiation was found to be maximum and hence the temperature difference was also maximum on that day. Though the incident radiation was maximum (876 W/m^2^) at 15 Hr, the observed temperature difference was found to be maximum at 14 Hrs when the incident radiation was 798 W/m^2^. This is because of the increased losses which reduced the heat gain of the collector. The average values of heat gain and efficiency for different days of study was recorded [[74], [75], [76], [77]]. Here the observed efficiency on April 20, 2021 when the incident radiation was maximum is found to be less, while it is maximum on April 17, 2021 when the incident radiation was 647.45 W/m^2^. This is because of the almost same heat gain on both days in spite of varying incident radiation. Since efficiency is the ratio of heat gain to the incident radiation, an increase in incident radiation reduces the efficiency for the same heat gain. In all the trials the simulated and observed results show satisfactory matching.

In the graphical representation, three different absorber collectors with solar incidence radiation are considered for both experimental and simulation analysis separately, if both experimental and simulated results are superimposed in single graph, it will be more complex to observe minor changes in the results. Since in the graph day-wise results are plotted this includes an average of all individual day results i.e., from 8 a.m. to 6 p.m.

**Fig. 19:**
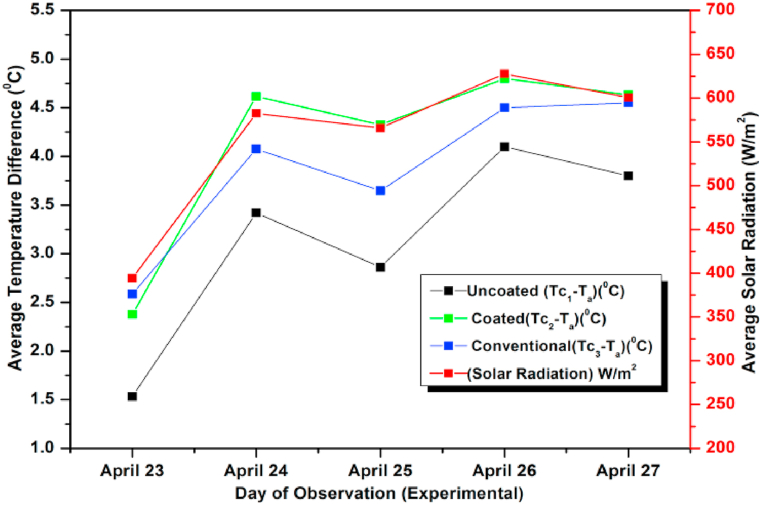
Variation of Day wise Average Temperature Difference (ΔT) (Observed) for 1 L/min.

**Fig. 20:**
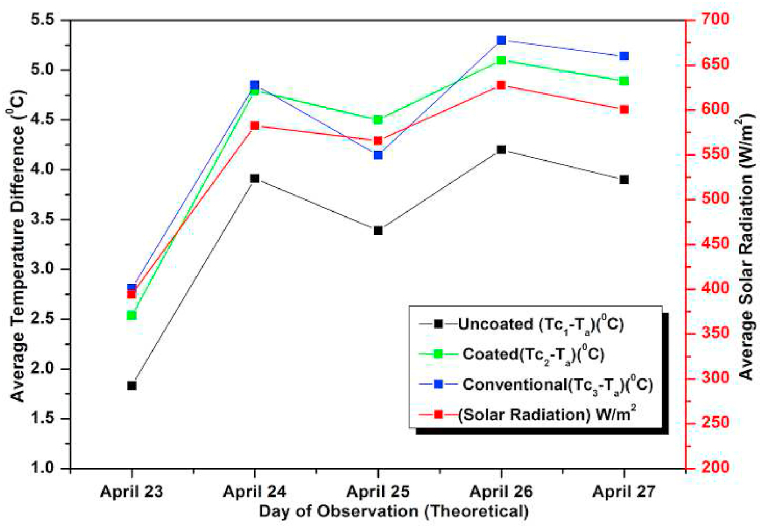
Variation of Daywise average temperature difference (ΔT) (simulated) for 1 L/min.

Next the flow rate was increased to 1 L/min. The experimental and simulated results are shown graphically (Fig. 19, Fig. 20). On April 26, 2021 the average incident radiation was found to be maximum and hence the temperature difference was also maximum on that day. As per the results the maximum incident radiation was at 13 h and the maximum observed temperature difference was also recorded at the same hour suggesting minor losses. The average values of heat gain and efficiency for different days of study was also recorded. As expected, the observed efficiency was found to be maximum on April 26, 2021 with a marginal difference on April 27, 2021. This is due to the marginal variation in losses on these days. Similar observation of getting highest lowest ΔT for the un-coated absorber and highest ΔT for the conventional absorber is seen here also [[78], [79], [80]]. The coated absorber's ΔT was in between the two, suggesting a slight reduction in performance compared to the conventional absorber. However, due to its affordability and easy availability, the coated pebble absorber is a viable alternative to the conventional absorber despite its slightly lower performance. In all the trials the numerical and observed results show satisfactory matching between the experimental and CFD results.

**Fig. 21:**
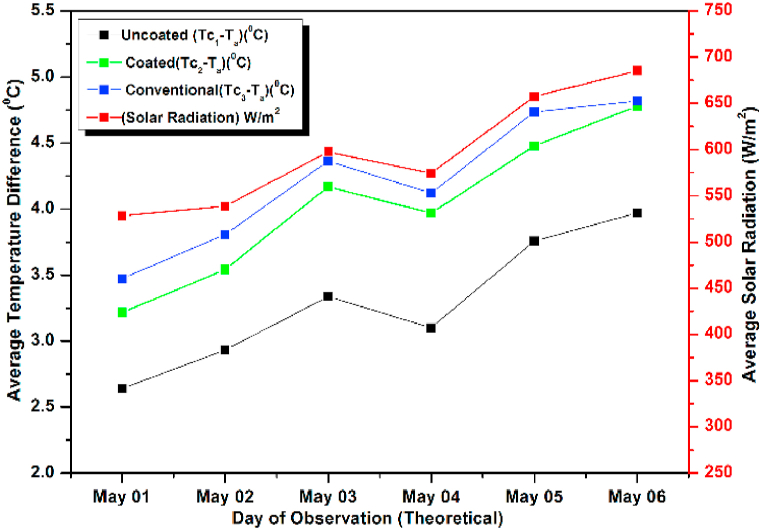
Variation of Day wise Average Temperature Difference (ΔT) (Observed) for 1.2 L/min.

**Fig. 22:**
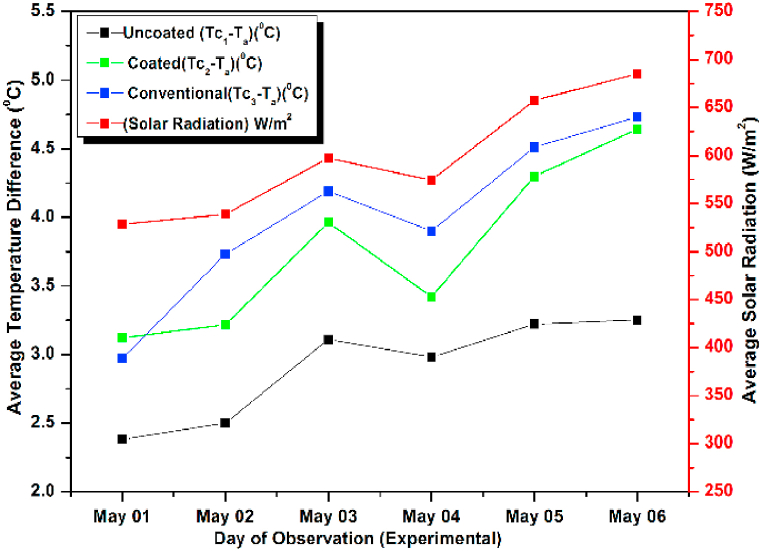
Variation of Day wise Average Temperature Difference (ΔT) (simulated) for 1.2 L/min.

Lastly the flow rate was increased to 1.2 L/min and study was conducted for six days from May 01, 2021 to May 06, 2021. The experimental and simulated results are shown graphically (Fig. 21, Fig. 22). On May 06, 2021 the incident radiation was found to be maximum and hence the temperature difference was also maximum on that day. As per the results the maximum incident radiation was at 14 h and the maximum observed temperature difference was also recorded at the same hour for the coated and conventional absorbers suggesting no losses, while for the un coated absorber the observed temperature difference decreased due to decreased heat gain [81]. The maximum observed efficiency was found to be on May 03, 2021 though, on this day, the heat gain is less due to low incident radiation, suggesting increased losses on May 06, 2021 for which the incident radiation was maximum. Here the losses may be due to the decreased ambient temperature or increased wind velocity. This suggests that the performance of the collectors depends not only on incident radiation but also on the corresponding losses. In all the trials the simulated and observed results show satisfactory matching. Since comparison was made for uncoated pebble absorber collectors, coated pebble absorber collectors, and conventional absorber collectors both numerically and experimentally. Each method has 4 different curves, if data obtained from both numerical and experimental methods are incorporated in the same figure, it will be 8 different curves where we cannot identify the variations in temperature difference and solar intensity.

The decrease in ΔT with an increase in flow rates can be attributed to the reduction in residence time of the fluid inside the collector. As the flow rate increases, the fluid spends less time inside the collector, reducing the heat transfer rate and resulting in a decrease in the ΔT. This trend is observed for all the days and for all the three types of absorbers - coated pebble, uncoated pebble, and copper absorber collectors. It is worth noting that although the coated pebble absorber collector exhibits a slightly lower temperature difference compared to the copper absorber collector, it is still a viable alternative due to its affordability and availability. The reduction in ΔT is marginal and can be compensated by other factors such as lower cost and ease of availability of the coated pebble absorber.

For all the flow rate it was observed that the average difference in efficiency between the coated and the conventional absorber collector is 5.8215 %, while difference between the coated and uncoated absorber collector is 15.686 %. Thus, it is very much evident that by replacing the conventional absorber by the proposed coated pebble absorber, the overall loss in efficiency is just 5.8215 %, but the advantages are enormous.

### 5.2 Structural analysis

Since in the present work Al/Cu tubes of the conventional collector are replaced by pebbles, the overall weight of the collector is bound to increase by more than 50 %. Hence a structural analysis was carried out to verify the design of the modified collector setup. Defining boundary conditions for the proposed collector involves, identifying the location of the boundaries (e.g., inlet valves, outlet valves, and walls) and supplying information at the boundaries. The Finite Element Method can handle any complex shape or geometry, as well as any material, under various boundary conditions. As mentioned earlier, the proposed experimental model is fixed at one end as shown in Fig. 6a, and to vary the tilt angle of the proposed collector, the header side is free to move and is provided with a nut and bolt arrangement, while the bottom side is permanently fixed to the frame.

**Fig. 23:**
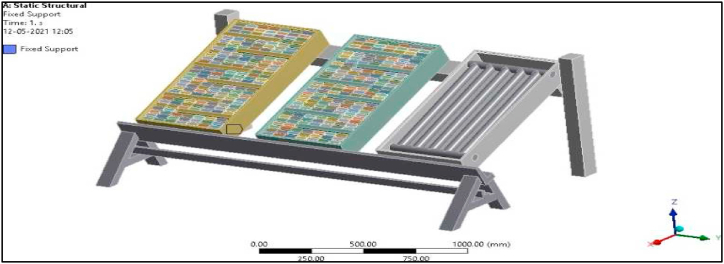
Proposed PASTC model fixed at one end.

**Fig. 24:**
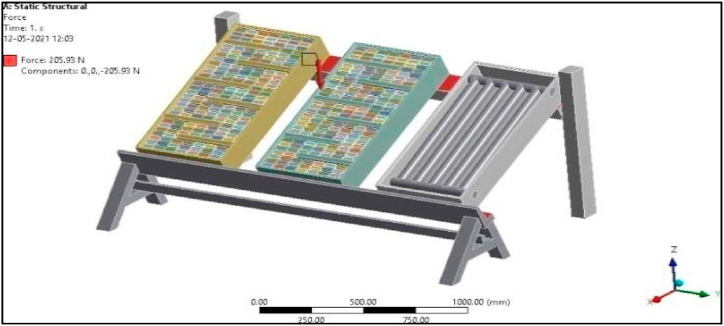
Force experienced by the PASTC set-up.

**Fig. 25:**
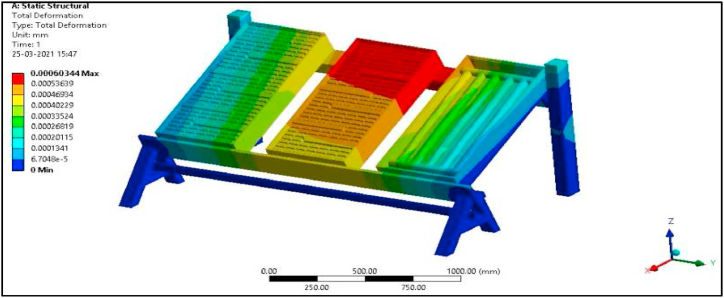
Total deformation of the set-up.

**Fig. 26:**
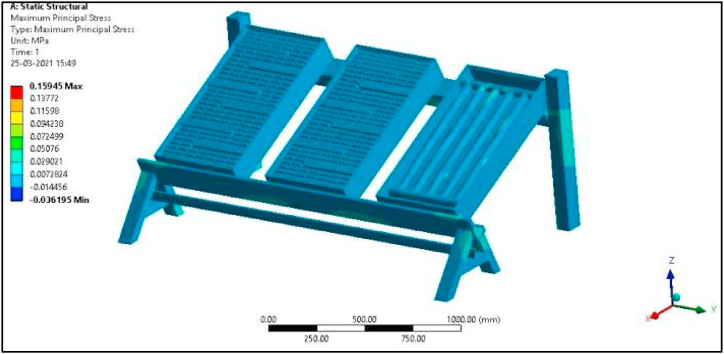
Maximum Principal Stress of the set-up.

The total force experienced by the PASTC is 205.93 N as shown in Fig. 23 and is well within the failure stress limit since the yield stress of mild steel is 250 MPa. The total deformation of the set-up is 0.00063 mm for the given boundary condition as shown in Fig. 24. Maximum principal stress of the set-up is 0.15945 MPa for the applied load condition as in Fig. 25. Thus, it is found that the designed structure is well within the failure limit and hence the design is very much safe (Fig. 26.).

**Fig. 27:**
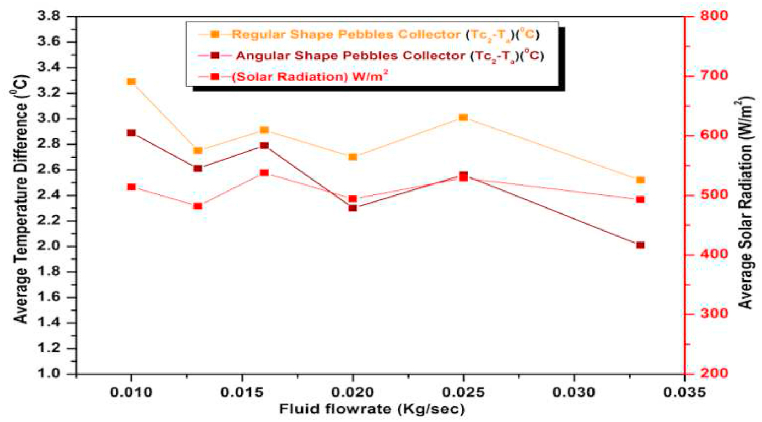
Variation of Average Temperature Difference (ΔT) (Observed) for Regular and Angular shape pebbles at varying flow rates.

**Fig. 28:**
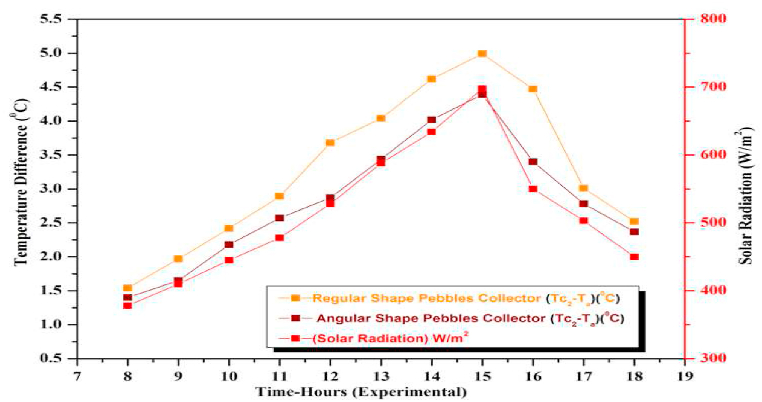
Hourly variation of Average Temperature Difference (ΔT) (Observed) for Regular and Angular shape pebbles on November 29, 2021.

The temperature variation of the heat absorbing medium depends on the orientation of the pebbles which in turn depends on the shape of the pebbles. The more the surface area of contact of the pebbles with the base plate more is the more heat loss resulting in a decrease in its temperature and hence the temperature of the heat absorbing medium. This will result in decreased heat gain which in turn decreases the efficiency of the collector. Thus, the shape of the pebbles which dictates the area of contact with the base plays a very important role in the performance of the collector and hence this study was undertaken. The study was done with two shapes viz. regular or spherical and irregular or angular. For each type, Average Temperature Difference, Heat gain were determined for varying flow rates at different days of the week and results are shown in Fig. 27, Fig. 28 at varying flow rates. Pebbles with spherical contours were found to be much more efficient than angular or irregular pebbles. This is because irregular pebbles tend to create gaps between themselves. These gaps are filled with air pockets and as air is an insulator, it results in reduced heat absorption by the absorber resulting in decreased performance of the collector. As per the results, the overall average values of efficiencies of the regular (spherical) and Irregular pebble absorbers are 62.038 % and 54.055 % respectively (calculated using equation (1)), suggesting that the regular pebbles are more efficient than the irregular pebbles.

**Fig. 29:**
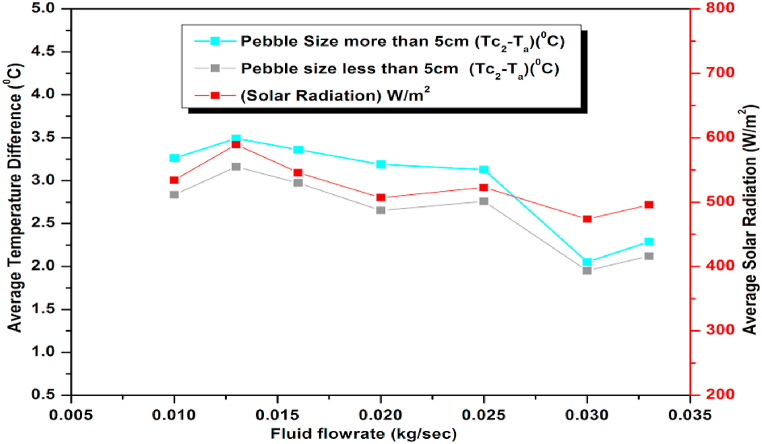
Variation of Average Temperature Difference (ΔT) (Observed) for Variable Pebbles Size and flow rates.

**Fig. 30:**
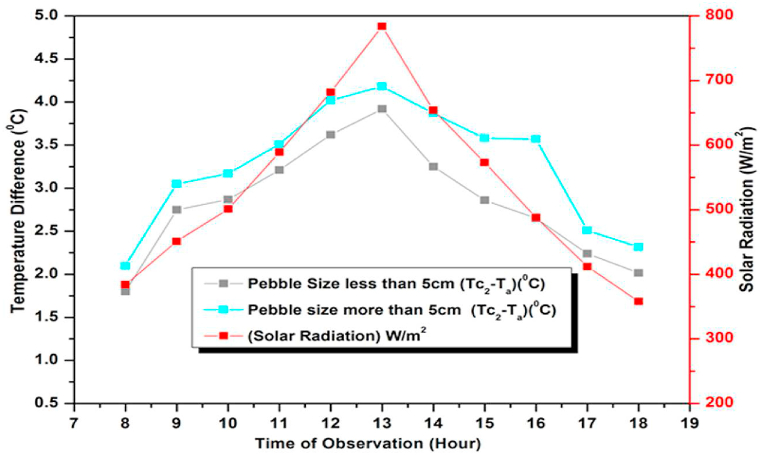
Hourly variation of Average Temperature Difference (ΔT) (Observed) for Variable Pebbles Size at different flow rates on December 15, 2021.

Since the rate of heat absorption depends on the surface area of the pebbles affecting the performance of the collector, study was also carried out to find the influence of the size (diameter) of the pebbles viz. <5 cms and >5 cms on the collector performance. For each type, both hourly and daily Average Temperature Difference and Heat gain were determined for varying flow rates. It was seen that; smaller size pebble absorbers perform less efficiently than their large-sized counterparts. Furthermore, it was observed that the variation in temperature for both sets of pebbles followed the incident radiation pattern both during hourly and daily studies (Fig. 29, Fig. 30) suggesting uniformity in the performance of both sets of pebbles.

## 6 Conclusion

In the present work, experimental and numerical analysis of the thermal performance of pebble solar thermal collectors (PSTC) is dealt with in detail using various parameters considered for the analysis on different days. The experimental set up with all the instrumentation was assembled and a series of tests were conducted by varying the operating variables viz. i) Flow rate of heat absorbing medium ii) Shape of the pebbles and iii) Size of the pebbles, simultaneously analysis was also carried out using CFD software.

The following conclusions can be drawn from the results.

• The study presented encouraging results with pebble absorber performing slightly lower than the conventional absorber. This authenticated the feasibility of the proposed collector and further study was carried out by designing and fabricating a collector made of coated, uncoated and conventional absorbers.
• For all the flow rates, it was observed that the average difference in efficiency between the coated and the conventional absorber collector is 5.8215 %, while difference between the coated and uncoated absorber collector is 15.686 %. Thus, it is very much evident that by replacing the conventional absorber with the proposed coated pebble absorber, the overall loss in efficiency is just 5.8215 %, but the advantages are enormous.
• Pebbles with spherical contours were found to be much more efficient than angular or irregular pebbles. This is because irregular pebbles tend to create gaps between themselves. These gaps are filled with air pockets and as air is an insulator, which results in reduced heat absorption by the absorber resulting in decreased performance of the collector.
• Smaller size pebble absorbers perform less efficiently than their large-sized counterparts.
• In the numerical analysis, the maximum relative error of 2.2 % and 2.4 % for average temperature difference was obtained for mass flow rates of 0.6 kg/s and 0.03 kg/s respectively. Therefore, these results are accurate enough and can be used for further design and analysis of the collector.
• Also, from the numerical analysis, it is evident that coated pebbles can be substituted in place of the metals in a conventional collector with a marginal (∼6 %) reduction in efficiency.

## Data availability statement

Data will be made available on request.

## CRediT authorship contribution statement

**N. Channa Keshava Naik:** Writing – original draft, Software, Methodology, Conceptualization. **R. Krishna Priya:** Writing – original draft, Software, Methodology, Conceptualization. **Ümit Ağbulut:** Writing – review & editing, Writing – original draft. **Ali Etem Gürel:** Writing – review & editing, Writing – original draft. **Saboor Shaik:** Writing – original draft, Methodology. **Ali Nasser Alzaed:** Writing – original draft, Methodology, Investigation, Conceptualization. **Mamdooh Alwetaishi:** Writing – original draft, Software, Methodology, Conceptualization. **Ahmad Aziz Alahmadi:** Writing – original draft, Software, Methodology.

## Declaration of competing interest

The authors declare that they have no known competing financial interests or personal relationships that could have appeared to influence the work reported in this paper.

## Nomenclature

**Symbols Abbreviations**
Qw: Total Energy (Heat) Gain (W)
I: Total Incident Radiation (W/m^2^)
ΔT: Temperature Difference (°C)
T_in_: Inlet Temperature (°C)
T_out_: Outlet Temperature (°C)
T_a_: Ambient Temperature (°C)
UB: Type B Uncertainty
C_p_: Specific Heat of fluid (J/Kg-°K)
SWH: Solar Water Heater
SFPC: Solar Flat Plate Collector
DVNR: Dynamically Vulcanized Natural Rubber
EPDM: Ethylene Propylene Diene Monomer
ETSC: Evacuated Tube Solar Collector
TPNR: Thermoplastic Natural Rubber
TRNSYS: Transient System Simulation Software
